# Origins and Previous Experiences from a Gender Perspective on the Perception of Pain in Nursing Students: Study Protocol

**DOI:** 10.3390/healthcare13182276

**Published:** 2025-09-11

**Authors:** Juan Manuel Pérez-Pozuelo, Almudena Crespo-Cañizares, Sonsoles Hernández-Iglesias, Nuria García-Magro, Ángel López-González, Victoria Lopezosa-Villajos, Miriam Hermida-Mota, Sagrario Gómez-Cantarino

**Affiliations:** 1 Faculty of Physiotherapy and Nursing, University of Castilla-La Mancha, 45071 Toledo, Spain; juanmanuel.perez5@alu.uclm.es (J.M.P.-P.); victoria.lopezosa@alu.uclm.es (V.L.-V.); miriam.hermida@alu.uclm.es (M.H.-M.); sagrario.gomez@uclm.es (S.G.-C.); 2 Health Sciences Faculty, Department of Nursery, University of Francisco de Vitoria, Pozuelo de Alarcón, 28223 Madrid, Spain; s.hernandez@ufv.es (S.H.-I.); nuria.garcia@ufv.es (N.G.-M.); 3 Faculty of Nursing, University of Castilla-La Mancha, 02006 Albacete, Spain; angel.lopez@uclm.es

**Keywords:** pain, pain management, non-pharmacological treatment, adolescent, gender perspective, students, nursing, health education, health culture

## Abstract

Background: The International Association for the Study of Pain (IASP) conceptualizes pain as a subjective experience, influenced by biopsychosocial factors, strongly related to the person’s environment and previous experience. It is necessary to understand painful experiences from birth and their influence on the self-perception of pain later in life. In addition, training competent health professionals to identify and treat pain becomes a priority. The main objective of the protocol is to describe the situations that influence pain perception. These influences are conceived from birth to adulthood, taking into account the gender perspective. Methods: This is a two-year exploratory mixed-methods educational intervention design, incorporating cross-sectional assessments at baseline. The research will be carried out in the academic community, including nursing students from two universities. The following will be carried out: (1) practical seminars (groups of 20–25) to increase future healthcare professionals’ awareness of pain-inducing procedures and critical thinking; (2) a peer-mentoring session, led by senior students, addressing pain research, its clinical impact, and strategies for improved pain management through theoretical and practical components; (3) supervised sessions, where students will learn and perform vital sign measurements (HR, SpO_2_), algometry, and the Cold Pressor Test (CPT) to assess pain perception, threshold, and tolerance, practicing in pairs; (4) a gender-specific questionnaire to evaluate students’ perceptions of pain, fostering reflection on gender differences in pain experiences. Discussion: The aim is to enhance knowledge about pain in future health professionals to increase their skills in the approach to pain. Conclusions: This study aims to promote formal academic contact between higher education students, promoting comprehensive care in the management of pain at different stages of life.

## 1. Introduction

The first formal definition of pain, proposed in 1979 by the IASP Subcommittee on Taxonomy and adopted by the IASP Council, conceptualized pain as “an unpleasant sensory and emotional experience associated with actual or potential tissue damage”, or described in such terms. This definition has been globally accepted by healthcare professionals and organizations, including the World Health Organization (WHO). Over the ensuing four decades, the scientific and clinical understanding of pain has expanded considerably [1,2,3]. This prompted the formation of a multinational IASP Presidential Task Force in 2018, composed of 14 experts in clinical and basic pain sciences, tasked with revisiting the original concept. In 2020, the IASP adopted a revised definition: “an unpleasant sensory and emotional experience associated with, or resembling that associated with, actual or potential tissue damage” [4]. Pain is recognized as a subjective phenomenon shaped by biological, psychological, and social factors, with nociception and pain defined as distinct processes [4,5]. This perspective underpins the concept of “total pain,” which integrates physical, emotional, social, and spiritual dimensions [6]. Importantly, pain can be experienced and expressed even without verbal communication [5]. Early exposure to painful stimuli can disrupt neurodevelopment and increase vulnerability to long-term behavioral and neurological consequences. In NICUs, neonates often undergo up to 300 painful procedures, leading to cumulative effects on neuropsychosocial development and future pain processing [7,8,9,10,11,12,13].

Adverse Childhood Experiences (ACEs) affect over 50% of children worldwide and are associated with heightened pain sensitivity, stress-related cortisol responses, and poorer treatment outcomes in adulthood [14,15,16,17,18,19,20,21,22]. Women with a history of childhood abuse report greater pain intensity and lower thresholds compared with those without such histories [14,23,24,25,26]. Recognizing pain relief as a fundamental human right, the UN underscores that no individual should suffer avoidable pain when effective treatments exist [27]. For neonates, reliable assessment remains challenging, but validated scales such as NFCS, N-PASS, COMFORTneo, NIPS, and FLACC enable consistent evaluation and guide effective management [7,28,29,30,31]. In the case of adolescence and young people, chronic pain during this stage has been shown to be associated with functional limitations and mental health problems in adulthood, underscoring the importance of addressing it at an early stage [32] Furthermore, in line with more contemporary perspectives, adolescence is increasingly recognized as extending up to the age of 24 years, as it represents a critical biological, psychological, and social transition into adulthood [33].

Once pain is assessed, healthcare professionals must judiciously select appropriate treatments. Pharmacological interventions in neonates must be approached with caution due to metabolic variability and the risk of adverse effects [34]. Opioids, while commonly used in NICUs, are associated with adverse neurodevelopmental outcomes [20,35,36,37,38,39] and organ toxicity—including in the kidneys, liver, and lungs—owing to the functional immaturity of these systems in neonates [7,40,41].

Given the limitations of pharmacological therapies, non-pharmacological interventions are an invaluable adjunct in neonatal pain management. These interventions, which modulate nociceptive transmission or activate descending inhibitory pathways, are associated with minimal side effects. Techniques include sucrose administration, skin-to-skin contact (kangaroo care), breastfeeding, non-nutritive sucking, facilitated tucking, music therapy, massage, environmental modifications, and aromatherapy [7,20]. Combining non-pharmacological methods with pharmacological treatments yields superior analgesic outcomes compared to either approach alone [20,42,43].

Despite advances in pediatric pain management, significant barriers persist in healthcare settings [44,45]. International evidence shows that pain education in nursing curricula remains insufficient, with persistent gaps in students’ knowledge and attitudes despite longstanding initiatives [46,47]. In Europe and Spain, nurses’ competencies in pain assessment and management remain suboptimal, although educational interventions—particularly in pediatrics—have proven effective in improving knowledge and practice [48,49,50]. These gaps highlight the need for innovative projects that incorporate pediatric pain and a gender perspective into nursing education [51]. In line with this, the IASP designated 2024 as the Global Year for Sex and Gender Disparities in Pain, underscoring the role of biological and sociocultural factors, with women showing greater vulnerability and higher prevalence of chronic pain [52,53,54,55,56,57,58,59]. Recent studies in fibromyalgia, a female-predominant condition, reveal this disproportionate burden, where women reported their pain being minimized or attributed to psychological causes. These findings indicate that disparities in pain are not solely biologically mediated but also reinforced by clinical practice, highlighting the urgent need for reforms in research and professional training [60].

The relationship between pain and sport warrants attention, as different sports entail varying risks of injury and pain, which in turn influence athletic participation and performance. The chronification of pain is determined not only by the initial injury but also by psychosocial and neurophysiological factors, the duration of sports practice, and the specific sport type [61]. A recent study in professional and semi-professional football in the United Kingdom further emphasized this interplay, highlighting the emotional impact of injuries and the fear of re-injury, which substantially affected recovery and rehabilitation outcomes. These findings underscore the critical role of psychosocial factors in shaping both the experience and management of sports-related pain [62].

The primary aim of this protocol is to examine the factors that influence pain perception from a gender perspective, while equipping future healthcare professionals with the knowledge to deliver effective and equitable pain management. The specific objectives are: (1) to assess nursing students’ prior knowledge regarding the relationship between gender and pain; (2) to raise awareness of the impact of routine clinical procedures and promote reflection on personal pain perception; (3) to provide theoretical training on pediatric pain within a cultural and gender framework; (4) to train the research team in non-pharmacological pain management using problem-based learning (PBL); and (5) to develop students’ skills in applying non-pharmacological strategies through active learning methodologies.

## 2. Materials and Methods

The research follows a mixed-methods educational intervention design, incorporating cross-sectional assessments at baseline. It will run across two academic years (2025–2026 and 2026–2027) with the collaboration of two partner institutions: (a) the Faculty of Physiotherapy and Nursing, Toledo Campus, University of Castilla-La Mancha, Spain, and (b) the Faculty of Health Sciences, Francisco de Vitoria University, Madrid, Spain. Both institutions have established a consortium grounded in their mutual commitment to advancing pediatric pain education and embedding a gender perspective within health sciences training.

The analytical plan will include descriptive and inferential statistics to evaluate differences between groups, as well as regression models to explore associations between gender, pain perception, and related variables. This approach will enable a more comprehensive analysis of how developmental, clinical, and psychosocial experiences interact with gender aspects in the perception and management of pain, thereby strengthening the methodological rigor and explanatory power of this study.

To frame the approach according to the needs of the project, the objectives are subdivided taking into account the following stakeholders:

(1) For students: to increase knowledge about total pain and pediatric pain; increase knowledge about non-pharmacological therapies for pediatric pain management and their practical application; to improve critical thinking; open up to sexual, social, gender and cultural diversity, and study how this impacts the perception of pain.
(2) For university lecturers: to increase skills/competences in non-pharmacological treatments; using innovative pedagogical approaches, in different modalities (face-to-face/distance learning).
(3) For universities involved in the project: to facilitate theoretical and practical studies in total pain and non-pharmacological management of pediatric pain; possibility of mobility and cooperation between partners; to develop innovative educational approaches (gamification); to produce face-to-face/distance learning formats; strengthen inter-university networks.
(4) For higher education degrees outside the project: to facilitate free access to distance learning formats on pain. This research will promote education at the international level in different higher education health courses at the member universities of this study.

Participants: participants will be individuals enrolled in higher education nursing programs, where the following inclusion criteria will be taken into account: (a) being enrolled in person in the third academic year; (b) having completed the Anatomy and Physiology course; (c) being over 18 years of age. Exclusion criteria: (a) students under the age of 18; (b) students enrolled in distance learning programs; and (c) students enrolled in the Erasmus program.

Sample: the sample size calculation was based on the main outcome of interest, namely the detection of gender-related differences in pressure pain thresholds assessed with algometry. Assuming a response proportion of 50% (*p* = 0.5), with a 95% confidence level and a margin of error of ±5%, the required minimum sample size was estimated at 152 participants, using the standard formula for finite populations.

Participants will be recruited from both partner universities (N_1_ and N_2_). While balanced recruitment across sites will be sought, the final number of students at each university will depend on actual participation rates.

Data Collection Instruments: the evaluation instruments are detailed in the following sections. It is important to note that the universities participating in the project enroll students from various regions of their respective countries. Consequently, the sociocultural characteristics of the sample are considered representative within each participating institution.

Quantitative Data Collection from Students: quantitative data will be gathered using a variety of instruments. The full questionnaire will be self-administered by students in paper format.

Ethical considerations: this study has received approval from the Ethics Committee of the University of Francisco de Vitoria (registration 46/2024), as well as from the relevant academic authorities of the collaborating universities. Teaching staff and students will receive an oral briefing about this study from the research team (one or two members at each university). Those who choose to participate will then be provided with written information. It is mandatory for both teachers and students to sign an informed consent form prior to taking part in this study.

The project will be carried out with participating students, organized in groups of 20–25 (N = 80 per university), developing the following outcomes:

Outcome 1.

A peer-mentoring session will be conducted in which participating students will attend a presentation designed and delivered by senior students (undergraduate or postgraduate) who are part of the research team at the participating universities. This session will aim to raise awareness of the importance of pain research as a central theme, in line with Objective 1. In addition, it will include the discussion of painful processes, their effects on both students and patients, their consequences, and the challenges of pain management, thereby addressing Objective 2, as these aspects are essential to improving care through more humane, holistic, and effective approaches.

Within this framework, the subsequent components should be understood as subthemes that expand on the main topic. The session will also explicitly integrate an analysis of the role of sex and gender in pain research, recognizing their critical influence on pain perception, expression, and management from a cultural perspective, in accordance with Objective 3 of this study. To achieve these goals, students will engage with scientific articles on pain and its multiple dimensions, develop infographics that synthesize key concepts, and actively participate in the class session itself, thereby reinforcing both theoretical knowledge and practical skills.

Furthermore, training will be provided to members of the research team at each of the participating universities, based on problem-based learning (PBL), thus fulfilling Objective 4.

Finally, through an activity that combines theoretical principles with practical applications, students will strengthen their understanding of pain from a non-pharmacological perspective and acquire the competencies necessary to act more effectively and safely for the benefit of patients. This training will be evaluated using a checklist to assess specific skills in the application of non-pharmacological techniques, thus achieving objective 5.

Outcome 2.

Theoretical and practical explanation of the techniques to be performed:

(1) Heart rate (HR) measurement technique and reference values in pediatrics.
(2) Pulse oximetry measurement technique as a non-invasive method for determining blood oxygen levels (O_2_) by emitting a beam of light through a pulsatile capillary. Students will be informed of the pediatric reference value for pulse oximetry (SpO_2_ > 93) [63,64].
(3) Algometry technique to determine the minimum pressure perceived by the participant as painful [65,66]. By gradually increasing the applied force on the skin, sensations progress from touch to pressure and finally to pain [67,68]. At specific measurement points (Table 1 and Table 2), students will rate pain intensity using a Visual Analogue Scale (VAS). The device will then be removed, and pain intensity will be recorded again using the VAS.

This technique serves as an indirect measure of the effect on sensory fibers at various sensitive points of the human body. A handheld algometer with an incremental scale of 0.1 N (Wagner Instruments, model FDIX), featuring a 1 cm diameter circular applicator, will be used. The applicator will be positioned perpendicularly to the skin, with pressure applied at approximately 1 kg/cm^2^ per second. Three measurements will be taken at 30 s intervals, and the average will be recorded as the pressure pain threshold. A 2 min rest will be allowed between measurement points to minimize sensitization effects. According to the study by Buskila [67], 9 tender points and 4 control points are identified, which will be used in the protocol as follows [67]:

These measurements will always be marked on the skin by the same investigator to ensure greater accuracy.

(4) Cold Pressor Test (CPT) [69] to assess pain threshold, tolerance, and intensity.

The test will be conducted using water at a temperature of 4–6 °C. Ice cubes will be removed before immersion to prevent direct skin contact. Participants will immerse their non-dominant forearm in the water, ensuring their fingertips touch the bottom of the container. Participants will be asked to verbally indicate when they first perceive discomfort by saying “it bothers me”, which will be recorded as their pain threshold. They will also rate the pain intensity using the VAS. Participants will then be instructed to withdraw their arm when they can no longer tolerate the pain, this point will be recorded as their pain tolerance. The pain intensity will again be rated on the VAS. If the participant does not withdraw their arm after 180 s, the test will be terminated—this is the maximum recommended duration, and participants will not be informed of this time limit in advance [69].

Outcome 3.

**Figure 1:**
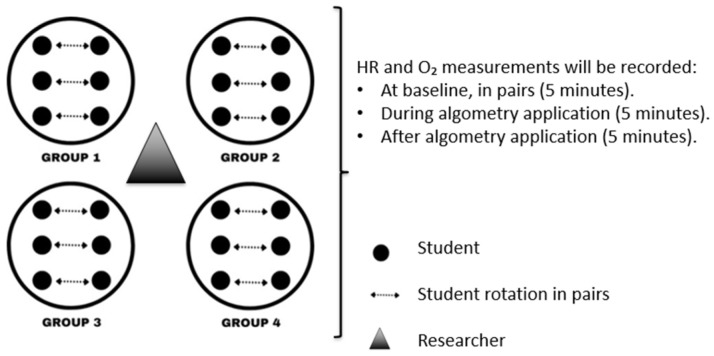
Six students are assigned to each group to perform the different measurements outlined in the protocol. Tasks are rotated among participants, ensuring that each student alternates roles (examiner and subject). All activities are conducted under the direct supervision of the research team.

All of these measurements explained in outcome 2 will be performed in groups of six students (Figure 1).

Each student will record their individual measurements including algometry and Cold Pressor Test on a designated form (Appendix A).

At the start of the intervention, a training video will be shown to teach students how to measure vital signs and use the algometer. Subsequently, each pair will have 60 min to perform measurements on both partners, followed by a 20 min break during which a gender-specific questionnaire will be completed. The total duration will be 80 min.

Outcome 4.

A gender-specific questionnaire will be completed to analyze students’ perceptions of painful processes (Appendix B).

## 3. Discussion

This project aims to create an open resource for use in both higher education and the community to promote a comprehensive and healthy understanding of pain and non-pharmacological pain management in pediatrics, taking into account gender perspectives. It also aims to promote formal academic contact between undergraduate students and the concept of pain and, consequently, to increase discussion on the subject in the training of future health professionals. This is important, given that, to date, many health professionals have only had contact with the concept through an informal curriculum [70,71]. In many places, competence in pain management is not a parameter analyzed in the licensing of health professionals. As a consequence of this slow adoption of the concept in the academic curriculum, professionals are trained who do not feel prepared to identify and treat cases of pain [72]. Thus, the project aims to train future health professionals with better preparation in this area.

The overall objective will be achieved through the achievement of the results presented in the intervention. The objectives will be achieved in a multicenter manner, which will allow for a broad discussion on the topic addressed, favoring the democratization of access to knowledge.

The results of this project will improve the development of knowledge about pain in academic settings, with an emphasis on nursing. In a context where persistent or chronic pain is a growing cause of disability-related morbidity worldwide [73,74], the results of this protocol aim to reduce barriers and challenges in teaching about pain: the slow adoption of pain content in the curriculum of HE courses and the lack of preparation of university professors in teaching the subject [73].

Currently, many health professionals lack adequate training in the concept of pain [71,73], which makes it difficult to pass on this knowledge to students. In this way, teachers participating in the protocol, by teaching classes on the subject and training in Outcomes 1 and 2, will be able to address what happened during the project and also in future occasions, contributing to the maintenance of the concept in academic curricula.

In addition to the general concept of pain, the concept of pediatric pain and pain in women will also be addressed. Although studies on this topic have increased since the 1980s, it is still clear that there are major gaps in the treatment of childhood pain, largely due to the need for holistic studies holistic studies on the subject [75]. In this way, by promoting educational interventions with both students and teachers, the results of the protocol will encourage further discussion of the subject in academia, with an emphasis on the non-pharmacological treatment of pediatric pain.

Another important analysis that will be carried out through the results of the protocol is the relationship between self-perception of health status and cultural differences between the groups involved, through the application of the form presented in outcome 4. In its definition of the concept of health, the World Health Organization (WHO) takes into account the physical, mental and social aspects of the individual [76]. In this sense, pain is an important factor in self-perception of health status, which is modulated by factors common to those present in the WHO definition [6,77,78]. With the data collected from the form, it will be possible to observe how this perception changes according to the student’s environment, even in a multicentric way, since we know that it originates through a cognitive process, which occurs from the acquisition of information, meanings, interpretations and representations, which are acquired from the sociocultural environment to which the individual belongs [78,79]. In this context, it is known that self-perception of health status can influence individuals’ daily practices, being associated with their choices for certain healthcare practices, including self-medication [79]. Thus, the objective is to discover how students define, perceive and alleviate their pain, relating it to: their previous experiences; sports practice, which is related to psychosocial aspects and varies the interpretation of pain; and gender, emphasizing the differences in the assessment of pain and its treatment from a gender perspective, focusing on self-perception and management of menstrual pain in women.

This study has limitations that should be acknowledged. First, as a protocol focused on nursing students, the results will not be generalizable to other populations or health professionals. The findings will reflect the particular context of undergraduate nursing education and may therefore be influenced by cultural, institutional, and curricular factors. Second, data collection relies on self-reported information, which may be subject to recall bias and social desirability bias. Third, a control group was not incorporated into this study, since the objective is not to determine the effectiveness of a single, tightly controlled intervention, but to examine the feasibility, acceptability, and educational impact of introducing innovative teaching approaches across both universities. Consequently, the design emphasizes ecological validity and wide participation rather than strict internal validity.

Despite these limitations, this study will provide novel insights into the role of gender and prior experiences in shaping the perception of pain among nursing students, and it may serve as a basis for future research in broader populations and with more diverse methodological approaches.

## 4. Conclusions

This protocol seeks to promote the concept of pain in the academic sphere, contributing to the training of future health professionals who are competent in the identification and treatment of pain, with an emphasis on neonatal and pediatric pain, and who analyze the perception of pain through a sociocultural and gender perspective, so that they can provide optimal treatment for different social groups. Finally, it is worth highlighting the project’s work in promoting health among students, so that they can increase their critical awareness of self-perception of pain, how it influences daily routines, and the differences that exist in its expression and approach among different individuals.

## Figures and Tables

**Table 1:** Sensitive points.

Sensitive Point	Anatomical Area	Side(s) Evaluated	Anatomical Description
Upper Trapezius	Trapezius	Right and left	Midpoint of the upper trapezius muscle (midway between the occiput and the acromion)
Occipital	Occipital	Right	Below the occipital prominence
Cervical Spine	Cervical Column	Right	Intertransverse space between C5 and C7
Second Rib	Second Rib	Right	On the superior surface, just lateral to the costochondral junction (sternum and costal cartilage)
Knees (medial side)	Knees	Right and left	In the medial adipose pad, over the medial collateral ligament (3 cm lateral to the medial border of the patella)
Lateral Epicondyle	Lateral Epicondyle	Right	2 cm distal (toward the wrist) to the lateral epicondyle of the humerus
Greater Trochanter	Greater Trochanter	Right	2 cm posterior to the greater trochanter of the femur (palpating the posterior border of the greater trochanter)

Source: According to the study by Buskila [67].

**Table 2:** Control points.

Control Point	Anatomical Description
Glabella	Area between the eyebrows, above the nasal bridge
Right Forearm	Distal third of the right forearm (the forearm is divided into thirds and a consistent point is selected for each participant within this area)
Right Knee	Lateral aspect of the knee (3 cm lateral to the midline of the patella)
Right Metatarsus	Shaft of the third metatarsal of the right foot

Source: According to the study by Buskila [67].

## Data Availability

No new data were created or analyzed in this study.

## Abbreviations

The following abbreviations are used in this manuscript:

IASP	International Association for the Study of Pain
WHO	World Health Organization
NICUs	Neonatal Intensive Care Unit
ACEs	Adverse Childhood Experiences
HE	Higher Education
HR	Heart Rate
VAS	Visual Analogic Scale
CPT	Cold Pressor Test

## Appendix A

**Table A1:** Measurement of Sensitive Points.

Anatomical Point	Side	Measurement 1 (N)	Pain (VAS1)	Measurement 2 (N)	Pain (VAS2)	Measurement 3 (N)	Pain (VAS3)	Mean (N)
**Upper Trapezius**	**Right**							
		HR:		HR:		HR:		
		O_2_:		O_2_:		O_2_:		
**Upper Trapezius**	**Left**							
		HR:		HR:		HR:		
		O_2_:		O_2_:		O_2_:		
**Occipital**	**Right**							
		HR:		HR:		HR:		
		O_2_:		O_2_:		O_2_:		
**Cervical Spine**	**Right**							
		HR:		HR:		HR:		
		O_2_:		O_2_:		O_2_:		
**Second Rib**	**Right**							
		HR:		HR:		HR:		
		O_2_:		O_2_:		O_2_:		
**Knee (Medial side)**	**Right**							
		HR:		HR:		HR:		
		O_2_:		O_2_:		O_2_:		
**Knee (Medial side)**	**Left**							
		HR:		HR:		HR:		
		O_2_:		O_2_:		O_2_:		
**Lateral Epicondyle**	**Right**							
		HR:		HR:		HR:		
		O_2_:		O_2_:		O_2_:		
**Greater Trochanter**	**Right**							
		HR:		HR:		HR:		
		O_2_:		O_2_:		O_2_:		
**Anatomic Point (2)**		**Measurement 1 (N)**		**Measurement 2 (N)**		**Measurement 3 (N)**		**Mean (N)**
**Glabella**								
		HR:		HR:		HR:		
		O_2_:		O_2_:		O_2_:		
**Right Forearm**								
		HR:		HR:		HR:		
		O_2_:		O_2_:		O_2_:		
**Right Knee (Lateral side)**								
		HR:		HR:		HR:		
		O_2_:		O_2_:		O_2_:		
**Right Metatarsus**								
		HR:		HR:		HR:		
		O_2_:		O_2_:		O_2_:		

*** Thermal Pain: (1) threshold; (2) tolerance.**

**Observations:**

**Time (s)**	**Pain (VAS)**
	
	

**Time (s)**	**Pain (VAS)**
	
	
Source: own elaboration based on Buskila research [67].	

## Appendix B

### Appendix B.1. Specific Questionnaire for Women

PAIN MANAGEMENT IN WOMEN.

BLOCK 9. PAIN PERCEPTION AND MENSTRUAL EXPERIENCE.

1. At what age did you start menstruating?

	<12 years		Between 12 and 14 years		Between 15 and 17 years		>18 years

2. Are your menstrual cycles regular?

      YES       NO

3. Indicate menstrual cycle frequency:

	<28 days		28–32 days
	>32 days		Irregular Cycles

4. Do you usually experience menstrual pain (dysmenorrhea)?

    YES     NO

*(If no, skip to question 10)*

5. Evaluate your menstrual pain on a scale from 1 to 10:

7. Which pattern describes your menstrual pain?
  Pain before bleeding, intense, then decreases
  Consistently intense pain throughout
  Mild but persistent pain
  Very mild pain that progressively disappears

8. Do you think your menstrual pain experience influences your perception of other types of pain?
      YES     NO
9. Do you think men tolerate pain better than women?
      YES     NO

BLOCK 10. MENSTRUAL PAIN MANAGEMENT.

10. What methods do you use to alleviate menstrual pain?
  Pharmacological analgesia
  Walking
  Relaxing baths
  Heat application
  Other: ________

11. Which drugs do you use? (multiple options possible):

Paracetamol Metamizol (nolotil ©) Dexketoprofeno (enantyum ©, enandol ©) Naproxeno (antalgin ©) Ibuprofeno Diclofenaco	Tramadol Codeína Dihidrocodeína	Have you ever needed oral contraceptives (OC) for menstrual pain? YES NO Are you currently taking OC? YES NO
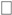

BLOCK 11. SOCIO-HEALTH ENVIRONMENT INFLUENCE.

1. Do you believe there is sufficient health education on menstrual pain prevention/management in your health/educational environment?
      YES     NO
2. Do you consider healthcare professionals important in managing menstrual pain?
      YES     NO
3. Evaluate on a Likert scale your satisfaction with the socio-health care received during menstruation:

### Appendix B.2. Specific Questionnaire for Men

PAIN MANAGEMENT IN MEN.

BLOCK 6. KNOWLEDGE ABOUT MENSTRUAL PAIN

1. Do you think students should receive more training on menstrual pain to better understand their patients?
      YES     NO
2. Menstrual pain can be disabling and significantly affect women’s daily activities.

	True
	False

3. Do you think men tolerate pain better than women?
      YES     NO
4. Indicate how important you think each of the following strategies is for managing menstrual pain:
  (a) Pharmacological therapy as a method to address menstrual pain and improve women’s quality of life
  (b) Non-pharmacological therapy (yoga, acupuncture, and relaxation techniques) as a method to address menstrual pain and improve women’s quality of life.

BLOCK 7. INFLUENCE OF THE SOCIO-HEALTH ENVIRONMENT ON THE PAINFUL MENSTRUAL EXPERIENCE

5. Do you consider that, in your healthcare and/or educational environment (high schools, universities, health centers, etc.), there is sufficient health education on the prevention and management of menstrual pain?
      YES     NO
6. Do you consider healthcare professionals important in addressing menstrual pain?
      YES     NO

BLOCK 8. MALE EXPERIENCE OF PAIN SELF-PERCEPTION

7. Do you think it is more common for men to ignore pain or delay going to the doctor compared to women?
      YES     NO
8. What do you think is the main reason men tend to avoid going to the doctor for pain?
  Fear of receiving bad news
  Lack of time
  Belief that “the pain will go away on its own without professional help”
  Preference for handling pain with home remedies or self-medication
9. What type of pain do you think is most prevalent in the male population?
  Back pain, especially lower back pain
  Muscle and joint pain
  Abdominal or groin pain
  Testicular pain
10. Indicate which factor you believe is most relevant in the difference in pain self-perception according to sex (choose only one option):
  The different hormones in the body influence the pain threshold and tolerance between men and women.
  The attitude toward pain in terms of openly expressing it influences the pain threshold and tolerance between men and women.
  Seeking medical help influences the pain threshold and tolerance between men and women.
11. Regarding testicular pain from trauma (blow), do you consider it a medical emergency?
      YES     NO
12. Regarding testicular pain from trauma, do you think there is a risk of serious complications such as a testicular hematoma, testicular torsion, or even rupture of the testicular membrane?
      YES     NO
13. If you have ever suffered testicular trauma, did you go to the doctor to assess the situation to prevent possible complications and/or treat the associated pain?
      YES     NO

### Appendix B.3. General Questionnaire

SOCIO-DEMOGRAPHIC DATA OF THE PARTICIPANT.

**Nursing Degree**		**Degree in Physiotherapy**
1st Year		
2nd Year		
3rd Year		
4th Year		

**Gender**	**Female**		**Male**		**Others**			
Age	18–25		26–33		33–40		>40	
University	UCLM (Toledo)		UCLM (Albacete)		UFV			

Do you work while studying?: YES     /NO
Do you have family responsibilities?: YES     /NO

PAINFUL EXPERIENCES AND PERSONAL PAIN MANAGEMENT TECHNIQUES.

BLOCK 1. PAIN AND PERINATAL EXPERIENCE.

1. In which week or month of gestation (WG) were you born?

	Less than 37 WG		Between 37–40 WG		More than 40 WG

2. If you were born before 37 weeks of gestation, how many weeks exactly were you born?
    ____________________________________________________
3. How much did you weigh at birth?
    ____________________________________________________
4. If you know your APGAR score at birth, indicate it numerically:________________________________________________________
5. Were you born via vaginal delivery (natural/eutocic)?
      YES     NO
6. If not, which method was used?
  Instrume-Forceps-assisted
  Spatula-assisted
  Vacuum-assisted
  Cesarean
  Other: ________

BLOCK 2. PREVIOUS PATHOLOGICAL EXPERIENCE.

7. Have you ever been admitted to the Neonatal or Pediatric Intensive Care Unit (NICU or PICU)?
      YES     NO
8. Have you ever been hospitalized?
      YES     NO
9. If yes, complete the following
  Age of admission: ________
  Length of stay (days): ________
  Reason for admission: ________
  Did you experience pain during hospitalization
        YES     NO
10. Do you have any disease that causes pain?
      YES     NO
    ____________________________________________________
    ____________________________________________________
    ____________________________________________________

11. If yes, which one?

BLOCK 3. HEALTHCARE ACCESS.

12. What type of healthcare center do you usually attend?
  Private                             Public

BLOCK 4. PAIN MANAGEMENT.

13. If you experience pain
  a. Do you seek healthcare assistance?
  b. Do you try to manage it at home by yourself?
14. Do you try to manage it at home by yourself?
  Rate your self-management capability from 1 to 10:
15. What measures do you usually take to relieve pain?
  Pharmacological analgesia
  Walking
  Relaxing baths
  Applying heat to the painful area (seed bags, hot water bottles)
  Other methods: ________
16. If using pharmacological measures, which drugs do you usually take? (select all that apply):

Paracetamol Metamizol (nolotil ©) Dexketoprofeno (enantyum ©, enandol ©) Naproxeno (antalgin ©) Ibuprofeno Diclofenaco	Tramadol Codeína Dihidrocodeína	Have you taken any of these drugs in the last 24 h? YES NO If yes, indicate which: ________________________
	Morfina Fentanilo Otros:	

BLOCK 5. SPORTS AND PAIN.

17. What type of physical activity do you regularly do?

	Grade 0: Hiking only, walking to university/work, household chores
	Grade 1: Minimum WHO activity guidelines (2.5–5 h/week moderate or 1.5–2 h/week intense activity, group sports)
	Grade 2: Local competitive level
	Grade 3: National competitive level
	Grade 4: International competitive level

Indicate which sport: ________________________________________________________
